# The genome sequence of horse-radish, *Armoracia rusticana* G.Gaertn., B.Mey. & Scherb. (Brassicales: Brassicaceae)

**DOI:** 10.12688/wellcomeopenres.27846.1

**Published:** 2026-09-18

**Authors:** Maarten J. M. Christenhusz, Ilia J. Leitch, Michael F. Fay, Royal Botanic Gardens Kew Genome Acquisition Lab, Plant Genome Sizing Collective, Darwin Tree of Life Barcoding collective, Wellcome Sanger Institute Tree of Life Management, Samples and Laboratory team, Wellcome Sanger Institute Scientific Operations: Sequencing Operations, Wellcome Sanger Institute Tree of Life Core Informatics team, Tree of Life Core Informatics collective, Darwin Tree of Life Consortium

**Affiliations:** 1 Royal Botanic Gardens Kew, Richmond, Surrey, England, UK; 2 Botanical Society of Britain and Ireland, St Albans, Hertfordshire, England, UK

**Keywords:** Armoracia rusticana, horse-radish, genome sequence, chromosomal, Brassicales

## Abstract

We present a genome assembly of *Armoracia rusticana* (horse-radish; Streptophyta; Magnoliopsida; Brassicales; Brassicaceae). The assembly consists of two haplotypes with total lengths of 605.90 megabases and 589.75 megabases. The haplotype 1 assembly and the haplotype 2 assembly each contain 16 chromosomal pseudomolecules; these account for 99.56% and 99.22% of their respective sequences. The mitochondrial genome assembly has a length of 316.39 kilobases and the plastid genome assemblies have lengths of 156.67 and 155.14 kilobases. Gene annotation of the haplotype 1 assembly by Ensembl identified 40,675 protein-coding genes. This assembly was generated as part of the Darwin Tree of Life project, which produces reference genomes for eukaryotic species found in Britain and Ireland.

## Species taxonomy

Eukaryota; Viridiplantae; Streptophyta; Streptophytina; Embryophyta; Tracheophyta; Euphyllophyta; Spermatophyta; Magnoliopsida; Mesangiospermae; eudicotyledons; Gunneridae; Pentapetalae; rosids; malvids; Brassicales; Brassicaceae; Cardamineae; *Armoracia*; *Armoracia rusticana* G.Gaertn., B.Mey. & Scherb. (NCBI:txid3704)

## Background

As part of the Darwin Tree of Life Project, a collaborative effort to sequence all named eukaryotic species in the Atlantic Archipelago of Britain and Ireland, we sequenced the genome of horse-radish, *Armoracia rusticana* G.Gaertn., B.Mey. & Scherb.

*Armoracia rusticana* is a member of Brassicaceae tribe Cardamineae. It is a glabrous perennial with deep, strong roots. It has a basal rosette of large undulate, obovate leaves with crenate margins. The stems grow to 1.5 m and have entire leaves. The inflorescence is a dense panicle of white, four-parted flowers.

Out of the three accepted species for the genus *Armoracia* G.Gaertn., B.Mey. & Scherb. ( POWO, 2026a), *A. rusticana* is thought to be native to southern European Russia, Ukraine and West Asia. However, the absence of documented extant wild populations may indicate that natural populations have gone extinct or that it exists only in cultivation and is derived from natural populations of other *Armoracia* species ( Sampliner & Miller, 2009).

The species occurs across the temperate Northern Hemisphere as an escape from cultivation ( POWO, 2026b). In Britain and Ireland, it is reported to be an archaeophyte (i.e., introduced before 1500). In cultivation, it spreads vegetatively, including when soil is moved as this can accidentally transport root fragments which can then grow and persist for many years. In England, it is widespread, only being absent from the highlands of south-western and northern England. In Wales, it is widespread in the lowlands, and in Scotland, it is most common in the lowlands between Edinburgh and Glasgow. In Ireland, it is widespread, but not as common as in mainland Britain ( Pearman & Walker, 2023). It is mostly found in wet grassy places and waste ground.

Horse-radish is estimated to have been cultivated for over 2000 years for its fleshy, pungent root which is widely used as a condiment, although it is believed to have initially been introduced into Britain and Ireland as a medicinal herb, and indeed it is still used as a traditional medicine in Central and Eastern Europe for a diversity of treatments ( Sampliner & Miller, 2009). Its antibacterial and anti-cancer properties are reported to be due to the presence of mustard oils such as allyl isothiocyanate ( Jimenez Negro *et al.*, 2022). This is produced when the plant is crushed or grated because it releases enzymes that break down the dominant glucosinolate sinigrin into allyl isothiocyanate ( Blažević *et al.*, 2020). By 1650 horse-radish had replaced *Lepidium latifolium* L. (dittander) as a vegetable cultivated for production of spicy sauces made from the root. Today, the pungent, fleshy root is grated and mashed and mixed with vinegar to produce commercial horse-radish sauce.

The sporophytic chromosome count is 2 *n* = 32 ( Manton, 1932), and the species is an allotetraploid with a basic chromosome number of *x* = 8 (e.g. Shen *et al.*, 2023). Most of the assembly is scaffolded into 16 chromosomal pseudomolecules, reflecting the gametophytic chromosome count of *n* = 16.

A chromosome-level genome assembly was previously reported for the cultivar ‘HD15’, grown in Hong Kong, China ( Shen *et al.*, 2023), and is publicly available through the National Genomics Data Center (GWHBJLW00000000.3; BioProject PRJCA009966), with assembly and annotation files also available on Figshare (doi:10.6084/m9.figshare.21780176.v2). This second high-quality genome assembly, from a naturalised specimen growing in England, provides a valuable complementary genomic resource for studying, for example, the origins of cultivated horse-radish and for comparative genomics of this and other crop plants in Brassicaceae.

**Figure 1.**
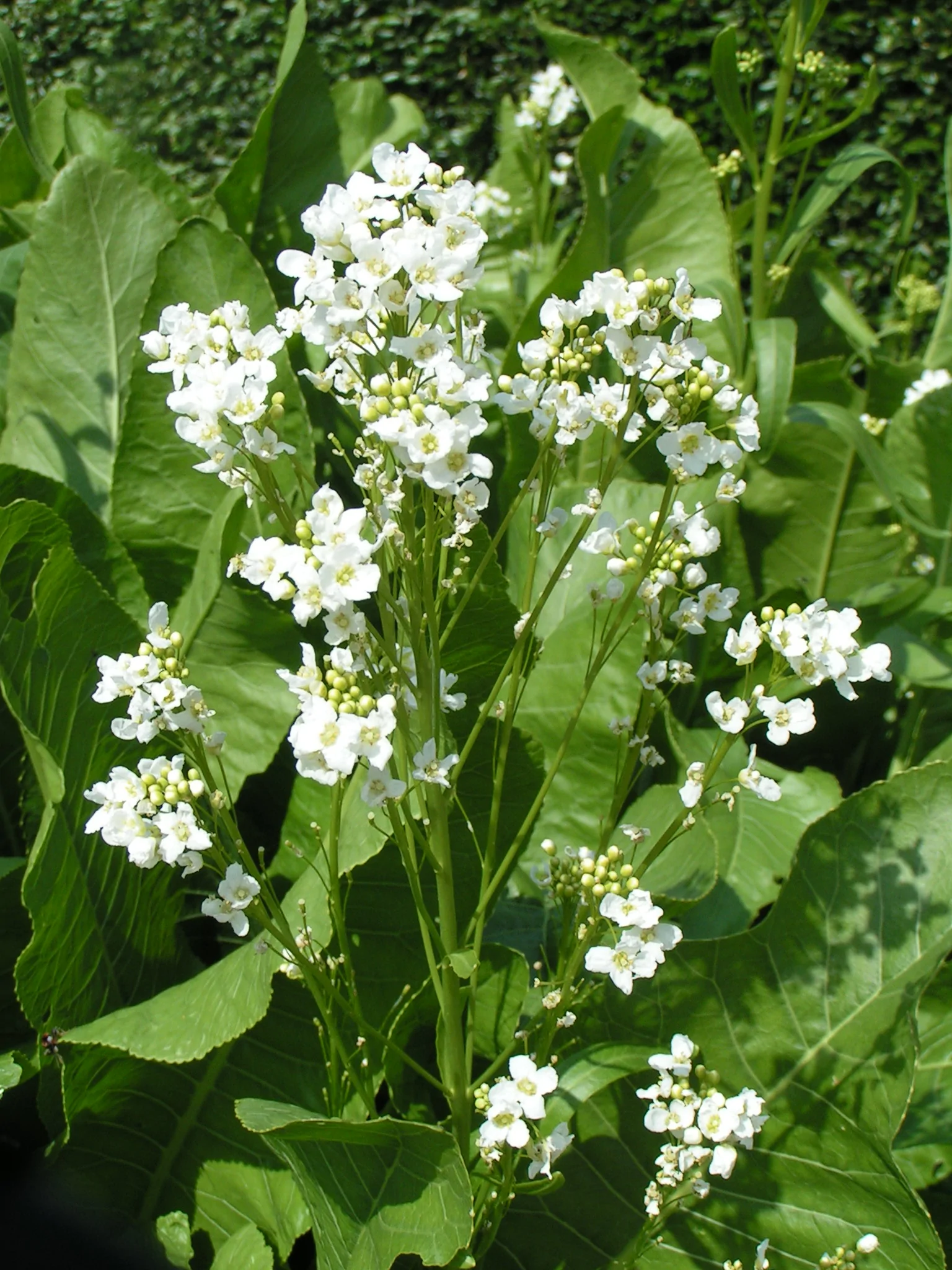
*A. rusticana* in Utrecht University Botanic Gardens, Netherlands. Photograph by Pethan.

## Methods

### Sample acquisition, flow cytometry and DNA barcoding

A specimen of *A. rusticana* (specimen ID KDTOL10362, ToLID ddArmRust1) was used for genome sequencing. It was collected from Kingston upon Thames, Surrey, UK (latitude 51.4359, longitude −0.3254) on 2021-08-17. The specimen was collected and identified by Maarten Christenhusz. The herbarium voucher for the sequenced plant (collector number MC9279) is deposited in the Herbarium, Royal Botanic Gardens, Kew (K), as specimen K001400892.

The genome size was estimated by flow cytometry following the ‘one-step’ method outlined in Pellicer *et al*. (2021) and using propidium iodide as the fluorochrome. The General Purpose Buffer (GPB) supplemented with 3% PVP was used for isolation of nuclei ( Loureiro *et al.*, 2007), and the internal calibration standard was *Petroselinum crispum* ‘Champion Moss Curled’ with an assumed 1C-value of 2,200 Mb ( Obermayer *et al.*, 2002).

The initial identification was verified by an additional DNA barcoding process according to the framework developed by Twyford *et al*. (2024). Part of the plant specimen was preserved in silica gel desiccant ( Chase & Hills, 1991). DNA extracted from the dried plant was amplified by PCR for standard barcode markers, with the amplicons sequenced and compared to public sequence databases including GenBank and the Barcode of Life Database (BOLD) ( Ratnasingham & Hebert, 2007). Following whole genome sequence generation, the relevant DNA barcode region was also used alongside the initial barcoding data for sample tracking at the WSI ( Twyford *et al.* , 2024). The standard operating procedures for Darwin Tree of Life barcoding are available on protocols.io.

### Nucleic acid extraction

High molecular weight (HMW) DNA extraction protocols developed by the Tree of Life Core Laboratory at the Wellcome Sanger Institute (WSI) are available on protocols.io ( Howard *et al.*, 2025). Tissue from the ddArmRust1 sample was weighed and triaged to select the appropriate extraction workflow. HMW DNA was prepared for three PacBio HiFi libraries: DTOL_RD13411303, DTOL14860866 and DTOL14860865. The concentration of the sheared and purified DNA was assessed with a NanoDrop spectrophotometer and a Qubit Fluorometer using the Qubit dsDNA High Sensitivity Assay kit. Fragment size distribution was evaluated by running the sample on the Femto Pulse system. The tissue input, HMW DNA extraction, downstream fragmentation and clean-up, and post-shearing quality-control measurements for each PacBio library are summarised in Table 1.

Library DTOL_RD13411303 was prepared from tissue BioSample SAMEA10369734; libraries DTOL14860866 and DTOL14860865 were prepared from tissue BioSample SAMEA10369736.

**Table 1.** DNA extraction and post-extraction processing for each PacBio library used for *A. rusticana*.

**PacBio library ID**	**Tissue preparation**	**HMW DNA extraction**	**Fragmentation and clean-up**	**Post-shearing QC**
DTOL_RD13411303	Leaf; cryoPREP	Plant Organic Extraction	Fragmentation: Megaruptor®3 for LI PacBio; clean-up: Automated SPRI	Not recorded
DTOL14860866	553.0 mg leaf; bead beating	Automated Sorbitol Washing + Plant Organic Extraction	Fragmentation: Megaruptor®3 for LI PacBio; clean-up: Automated SPRI	Qubit 46.6 ng/μL; fragment size 15.6 kb
DTOL14860865	553.0 mg leaf; bead beating	Automated Sorbitol Washing + Plant Organic Extraction	Fragmentation: Megaruptor®3 for LI PacBio; clean-up: Automated SPRI	Qubit 51.4 ng/μL; fragment size 16.5 kb

### PacBio HiFi library preparation and sequencing

Library preparation and sequencing took place in the WSI Scientific Operations core. Libraries DTOL_RD13411303, DTOL14860866 and DTOL14860865 were prepared with the SMRTbell Prep Kit 3.0 (Pacific Biosciences) according to the manufacturer’s instructions. The kit includes reagents for end repair/A-tailing, adapter ligation, post-ligation SMRTbell bead clean-up, and nuclease treatment. Size selection and clean-up were performed using diluted AMPure PB beads (Pacific Biosciences). DNA concentration was quantified with a Qubit Fluorometer v4.0 (Thermo Fisher Scientific) and the Qubit 1X dsDNA HS assay kit. Final library fragment size was assessed with the Agilent Femto Pulse Automated Pulsed Field CE Instrument (Agilent Technologies) using the gDNA 55 kb BAC analysis kit.

Library DTOL_RD13411303 was sequenced using the Sequel IIe system (Pacific Biosciences, California, USA). The concentration of the library loaded onto the Sequel system was in the range 40–135 pM. SMRT Link software, a PacBio web-based workflow manager, was used to set up and monitor the run, and to perform primary and secondary analysis of the data upon completion.

Libraries DTOL14860866 and DTOL14860865 were sequenced on a Revio instrument (Pacific Biosciences, California, USA). The prepared libraries selected for multiplexing were pooled based on genome size, library molarity, and the intended plex level. Primers were annealed and polymerases were bound to generate circularised complexes, following the manufacturer’s instructions. Complexes were purified using SMRTbell beads, diluted to the Revio loading concentration, and spiked with a Revio sequencing internal control. The pooled libraries were sequenced on a Revio 25M Plex SMRT cell in a 2-plex run. SMRT Link software (Pacific Biosciences), a web-based workflow manager, was used to configure and monitor the runs and to carry out primary and secondary data analysis.

### Hi-C

***Sample preparation and crosslinking***

Hi-C data were generated from the ddArmRust1 leaf sample using the Arima-HiC v2 kit (Arima Genomics). Tissue was finely ground using the Covaris cryoPREP Dry Pulverizer (Covaris), and then subjected to nuclei isolation. Nuclei were isolated using a modified protocol based on the Qiagen QProteome Cell Compartment Kit (Qiagen), in which only the Lysis and CE2 buffers were used, with QIAshredder spin columns. After isolation, nuclei were fixed using formaldehyde to a final concentration of 2% to crosslink the DNA. The crosslinked DNA was then digested and biotinylated according to the manufacturer’s instructions. A clean-up step was performed with SPRIselect beads before library preparation. DNA concentration was quantified using the Qubit Fluorometer v4.0 (Thermo Fisher Scientific) and the Qubit HS Assay Kit, following the manufacturer’s instructions.

***Hi-C library preparation and sequencing***

Biotinylated DNA constructs were fragmented using a Covaris E220 sonicator and size-selected to 400–600 bp using SPRISelect beads. DNA was enriched with Arima-HiC v2 kit Enrichment beads. End repair, A-tailing, and adapter ligation were carried out with the NEBNext Ultra II DNA Library Prep Kit (New England Biolabs), following a modified protocol where library preparation occurs while DNA remains bound to the Enrichment beads. Libraries were amplified with KAPA HiFi HotStart mix and a custom Unique Dual Index (UDI) barcode set (Integrated DNA Technologies). Depending on sample concentration and biotinylation percentage determined at the crosslinking stage, libraries were amplified with 10–16 PCR cycles. Post-PCR clean-up was performed with SPRISelect beads. Libraries were quantified using the AccuClear Ultra High Sensitivity dsDNA Standards Assay Kit (Biotium) and a FLUOstar Omega plate reader (BMG Labtech).

Prior to sequencing, libraries were normalised to 10 ng/μL. Normalised libraries were quantified again to create equimolar and/or weighted 2.8 nM pools. Pool concentrations were checked using the Agilent 4200 TapeStation (Agilent) with High Sensitivity D500 reagents before sequencing. Libraries were sequenced on the Illumina NovaSeq 6000, generating paired-end 150-bp reads.

### Genome assembly

Prior to assembly of the PacBio HiFi reads, a database of *k*-mer counts ( *k* = 31) was generated from the filtered reads using FastK. The *k*-mer frequency distribution was analysed with GenomeScope (version 2.1.0) ( Ranallo-Benavidez *et al.*, 2020), providing fitted point estimates of genome size, heterozygosity and repeat content.

The HiFi reads were assembled using Hifiasm in Hi-C phasing mode ( Cheng *et al.*, 2021, 2022), producing two haplotypes. Hi-C reads ( Rao *et al.* , 2014) were mapped to contigs using bwa-mem2 ( Vasimuddin *et al.*, 2019). Contigs were further scaffolded with Hi-C data in YaHS ( Zhou *et al.*, 2023), using the --break option for handling potential misassemblies. The scaffolded assemblies were evaluated using Gfastats ( Formenti *et al.* , 2022), BUSCO ( Manni *et al.*, 2021) and MerquryFK ( Rhie *et al.*, 2020). The organelle genomes were assembled using OATK ( Zhou *et al.*, 2025).

### Assembly curation

The assembly was decontaminated using the Assembly Screen for Cobionts and Contaminants ( ASCC) pipeline. The flat files and maps for curation were generated with TreeVal. Manual curation was conducted primarily in PretextView and HiGlass ( Kerpedjiev *et al.* , 2018). Scaffolds were visually inspected and corrected as described by Howe *et al*. (2021). The two haplotype assemblies were combined for curation. Manual corrections included 24 breaks and 210 joins. This reduced the scaffold count by 96.8%, reduced the scaffold N50 by 8.5%, and reduced the total assembly length by 11.5%. The curation process is described at sanger-tol/curation-resources. A Hi-C contact map of the haplotype 1 assembly was generated with PretextSnapshot.

### Assembly quality assessment

The MerquryFK tool ( Rhie *et al.*, 2020) was run in a Singularity container ( Kurtzer *et al.*, 2017) to evaluate *k*-mer completeness and assembly quality for both haplotypes using the *k*-mer database ( *k* = 31) computed prior to genome assembly. The analysis outputs included assembly QV scores and completeness statistics.

The haplotype 1 assembly was assessed using BlobToolKit ( Challis *et al.*, 2020). PacBio reads were mapped to the haplotype 1 assembly to derive sequence coverage, and BUSCO ( Manni *et al.*, 2021) was used to assess gene-set completeness. DIAMOND BLASTp searches of extracted BUSCO proteins and DIAMOND BLASTx searches of assembly sequences against UniProt Reference Proteomes ( Buchfink *et al.*, 2021), followed by BLASTn searches of sequences without DIAMOND BLASTx hits against the NCBI nt database ( Altschul *et al.*, 1990), supported taxonomic assignment. Coverage, sequence composition, taxonomic assignment and BUSCO results were integrated into a BlobDir for interactive inspection and visualisation. The snail and blob plots were rendered locally from this hosted BlobDir using BlobTk v0.8.3. The scaffold-length radial scale function for the snail plot was set to linear ( Challis & Blaxter, 2026). The blob plot used circular markers with area proportional to scaffold length ( Challis *et al.*, 2020).

## Genome sequence report

### Sequence data

The genome of a specimen of *A. rusticana* was sequenced using Pacific Biosciences single-molecule HiFi long reads, generating 46.45 Gb (gigabases) from 3.32 million reads. We assembled the genome from these reads. GenomeScope analysis using a *p* = 2 model gave a *k*-mer-based haploid genome size estimate of 667.54 Mb, with heterozygosity of 0.74% and repeat content of 52.56%; the full-model fit was 96.66% (Figure 2). Using flow cytometry, the genome size (1C‐value) of the sample was estimated to be 0.78 pg, equivalent to 770.00 Mb. These estimates guided expectations for the assembly. The sequencing data provided approximately 66× coverage of the haplotype 1 assembly. Hi-C sequencing produced 220.81 Gb from 731.17 million read pairs, which were used to scaffold the haplotype 1 assembly. Table 2 summarises the specimen and sequencing details.

**Figure 2.**
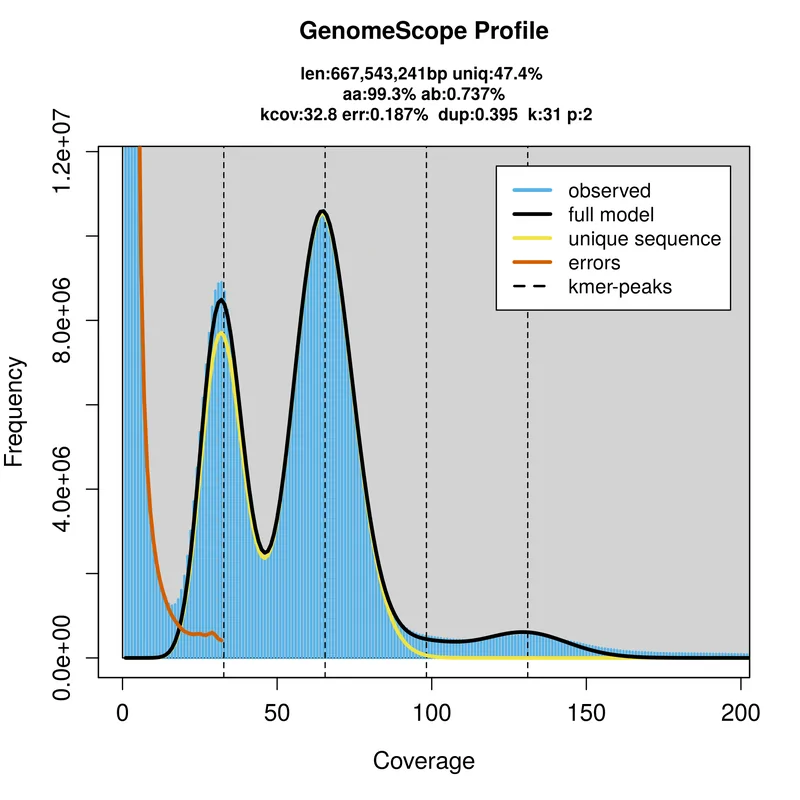
GenomeScope model fitted to the *k*-mer frequency distribution. The plot shows the observed and modelled *k*-mer spectra used to estimate genome size, heterozygosity and repeat content from unassembled sequencing reads.

**Table 2.** Specimen and sequencing data for *A. rusticana* (BioProject PRJEB80112)

**Platform**	**PacBio HiFi**	**Hi-C**
**ToLID**	ddArmRust1	ddArmRust1
**Specimen ID**	KDTOL10362	KDTOL10362
**BioSample (source individual)**	SAMEA10369658	SAMEA10369658
**BioSample (tissue)**	SAMEA10369734; SAMEA10369736	SAMEA10369734; SAMEA10369736
**Tissue**	leaf	leaf
**Instrument**	Sequel IIe (1 run); Revio (2 runs)	Illumina NovaSeq 6000
**Library ID & run accession(s)**	DTOL_RD13411303: ERR13698296; DTOL14860866: ERR13698295; DTOL14860865: ERR13698294	ERR13670059; ERR13670058
**Read count total**	3.32 million reads	731.17 million read pairs
**Base count total**	46.45 Gb	220.81 Gb

### Assembly statistics

The genome was assembled into two haplotypes using Hi-C phasing. The haplotype 1 assembly was curated to chromosome level, and haplotype 2 was also scaffolded to chromosome level. The haplotype 1 assembly, which is proposed as the reference assembly for this species, has a total length of 605.90 Mb in 62 scaffolds (excluding organelle sequences), with 372 gaps, and a scaffold N50 of 35.05 Mb (Table 3). The haplotype 1 assembly has a scaffold auN of 38.5 Mb.

**Table 3.** Genome assembly statistics for *A. rusticana*

**Genome assembly**	**Haplotype 1**	**Haplotype 2**
**Assembly name**	ddArmRust1.hap1.1	ddArmRust1.hap2.1
**Assembly accession**	GCA_965620475.1	GCA_965620565.1
**Assembly level**	chromosome	chromosome
**Span (Mb)**	605.90	589.75
**Number of chromosomes**	16	16
**Number of contigs**	434	455
**Contig N50 (Mb)**	3.3	3.16
**Number of scaffolds (excluding organelle sequences)**	62	114
**Scaffold N50 (Mb)**	35.05	35.83
**Scaffold auN (Mb)**	38.5	36.8
**Longest scaffold length (Mb)**	57.02	48.38
**Organelles**	Mitochondrial genome: 316.39 kb; Plastid genomes: 156.67 and 155.14 kb	-

Most of the haplotype 1 assembly sequence (99.56%) was assigned to 16 chromosome-level scaffolds. These chromosome-level scaffolds, confirmed by Hi-C data, are named according to size (Figure 3; Table 4). This genome has been assembled using PacBio and HiC data and phased. The result is two curated haplotypes.

During genome curation we noted that the exact order and orientation of the contigs on chromosome 1 (9.3–30.2 Mbp) are unknown. Multiple rearrangements (inversions, translocations) were observed in the region on chromosome 8 (14.1–19.7 Mbp). A haplotypic inversion was observed in the region on chromosome 2 (12.6–14.7 Mbp), chromosome 3 (26.5–28.6 Mbp), chromosome 7 (13.7–15.3 Mbp), chromosome 10 (10.6–11.8 Mbp), chromosome 11 (3.5–5.1 Mbp) and chromosome 16 (8.1–9.3 Mbp and 17.0–19.7 Mbp).

The mitochondrial genome (length 316.39 kb, OZ278782.1) and two plastid genomes (lengths 156.67 kb [OZ278783.1] and 155.14 kb [OZ278784.1]) were also assembled. These sequences are included as contigs in the multifasta file of the haplotype 1 genome submission and as standalone records.

**Figure 3.**
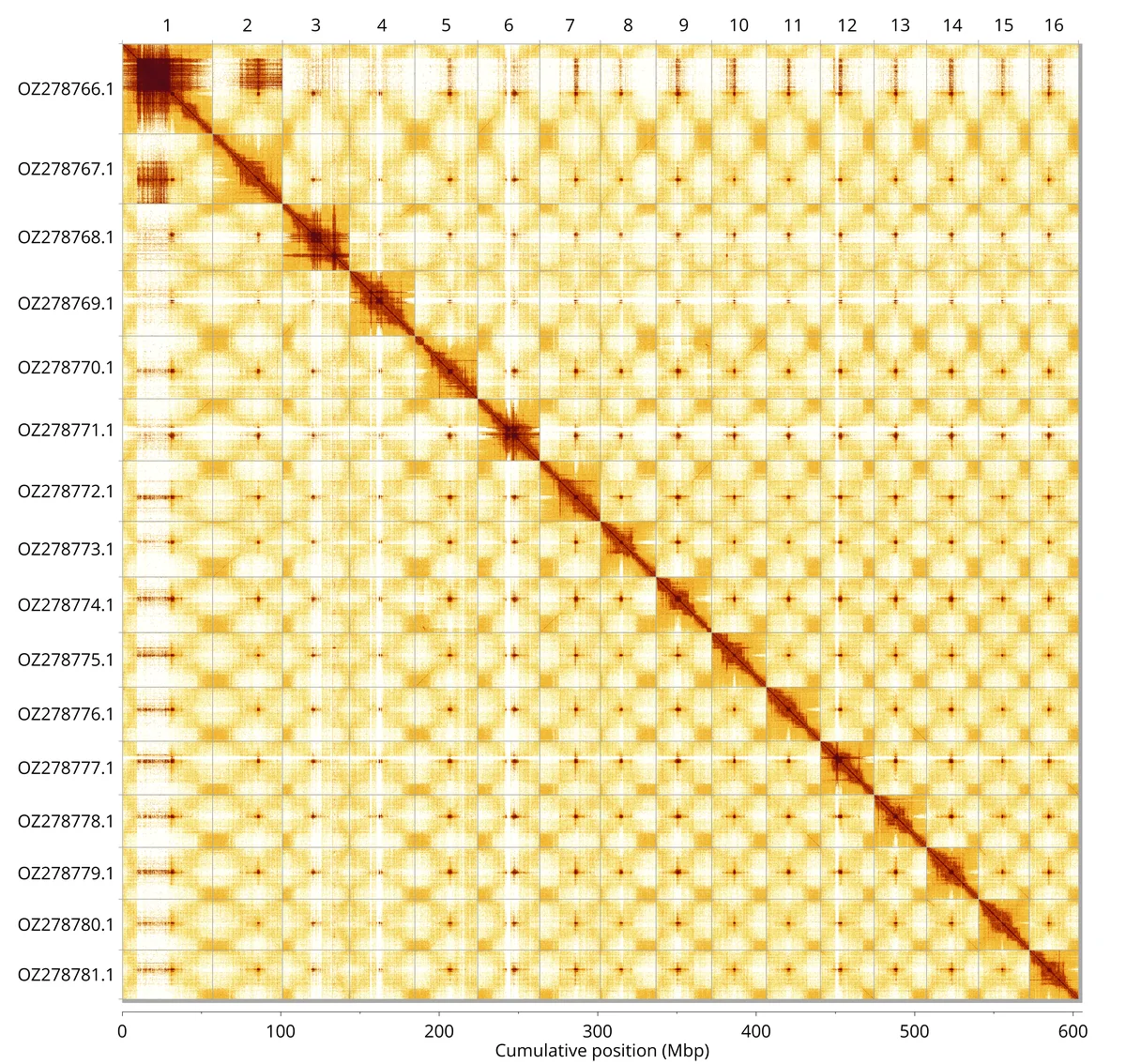
Hi-C contact map of the *A. rusticana* haplotype 1 assembly. Assembled chromosomes are shown in order of size and labelled along the axes, with a megabase scale shown below. The plot was generated using PretextSnapshot.

**Table 4.** Chromosomal pseudomolecules in the haplotype 1 genome assembly of *A. rusticana* ddArmRust1 (haplotype 2 also at chromosome level)

**INSDC accession**	**Molecule**	**Length (Mb)**	**GC%**
OZ278766.1	1	57.02	42
OZ278767.1	2	43.95	37.50
OZ278768.1	3	42.45	37.50
OZ278769.1	4	41.06	37.50
OZ278770.1	5	39.66	37
OZ278771.1	6	39.16	37
OZ278772.1	7	38.30	37
OZ278773.1	8	35.05	36.50
OZ278774.1	9	35.04	37.50
OZ278775.1	10	34.51	37.50
OZ278776.1	11	34.05	37
OZ278777.1	12	33.94	37
OZ278778.1	13	33.05	37
OZ278779.1	14	32.77	37
OZ278780.1	15	32.17	37
OZ278781.1	16	31.03	37

### Assembly quality metrics

For the haplotype 1 assembly, the estimated QV is 66.3, and for the haplotype 2 assembly, 65.2. When the two haplotypes are combined, the assembly achieves an estimated QV of 65.7. The *k*-mer completeness is 81.70% for the haplotype 1 assembly, 81.61% for the haplotype 2 assembly, and 97.61% for the combined haplotypes (Figure 4).

BUSCO analysis using the brassicales_odb10 reference set ( *n* = 4,596) identified 99.7% of the expected gene set (single = 28.9%, duplicated = 70.8%) in the haplotype 1 assembly. For the haplotype 2 assembly, BUSCO v.5.8.3 analysis identified 99.7% of the expected gene set (single = 29.1%, duplicated = 70.6%).

**Figure 4.**
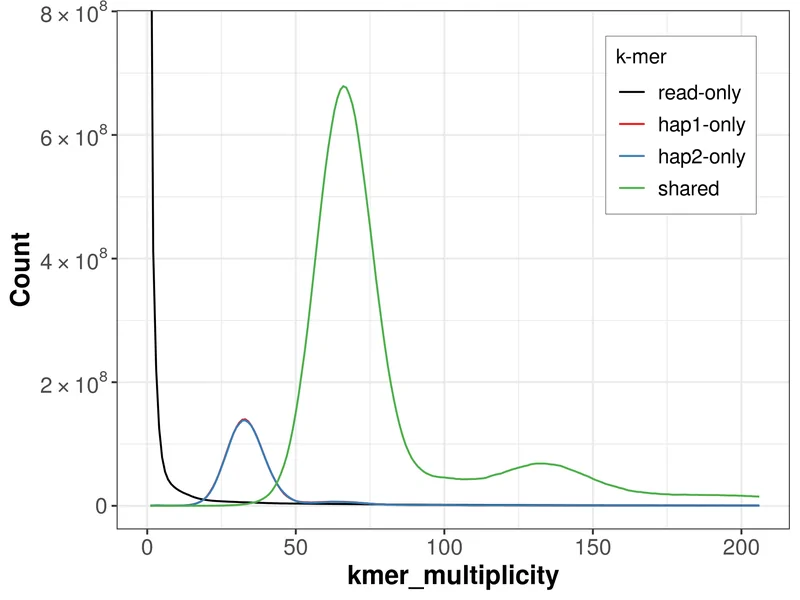
Evaluation of *k*-mer completeness using MerquryFK. This plot illustrates the recovery of *k*‐mers from the original read data in the haplotype 1 assembly, the haplotype 2 assembly and the combined haplotypes. The horizontal axis represents *k*‐mer multiplicity, and the vertical axis shows the number of *k*‐mers. The black curve represents *k*‐mers that appear in the reads but are not assembled, while the remaining curves correspond to *k*‐mers recovered in the assemblies shown.

The snail plot in Figure 5 provides a summary of assembly contiguity, cumulative scaffold count, sequence composition and BUSCO completeness for the haplotype 1 assembly ( Challis & Blaxter, 2026). The relative sizes of the longest scaffold, scaffold N50 and N90, together with the dark-grey scaffold-length distribution, show how much of the assembly is contained in long scaffolds. The outer composition track shows variation in GC, AT and N content, while the central track shows the cumulative scaffold count and the BUSCO inset summarises gene-set completeness.

The blob plot in Figure 6 displays scaffolds from the haplotype 1 assembly by GC proportion and base coverage. Marker area is proportional to scaffold length, and colour indicates taxonomic assignment ( Challis *et al.*, 2020).

**Figure 5.**
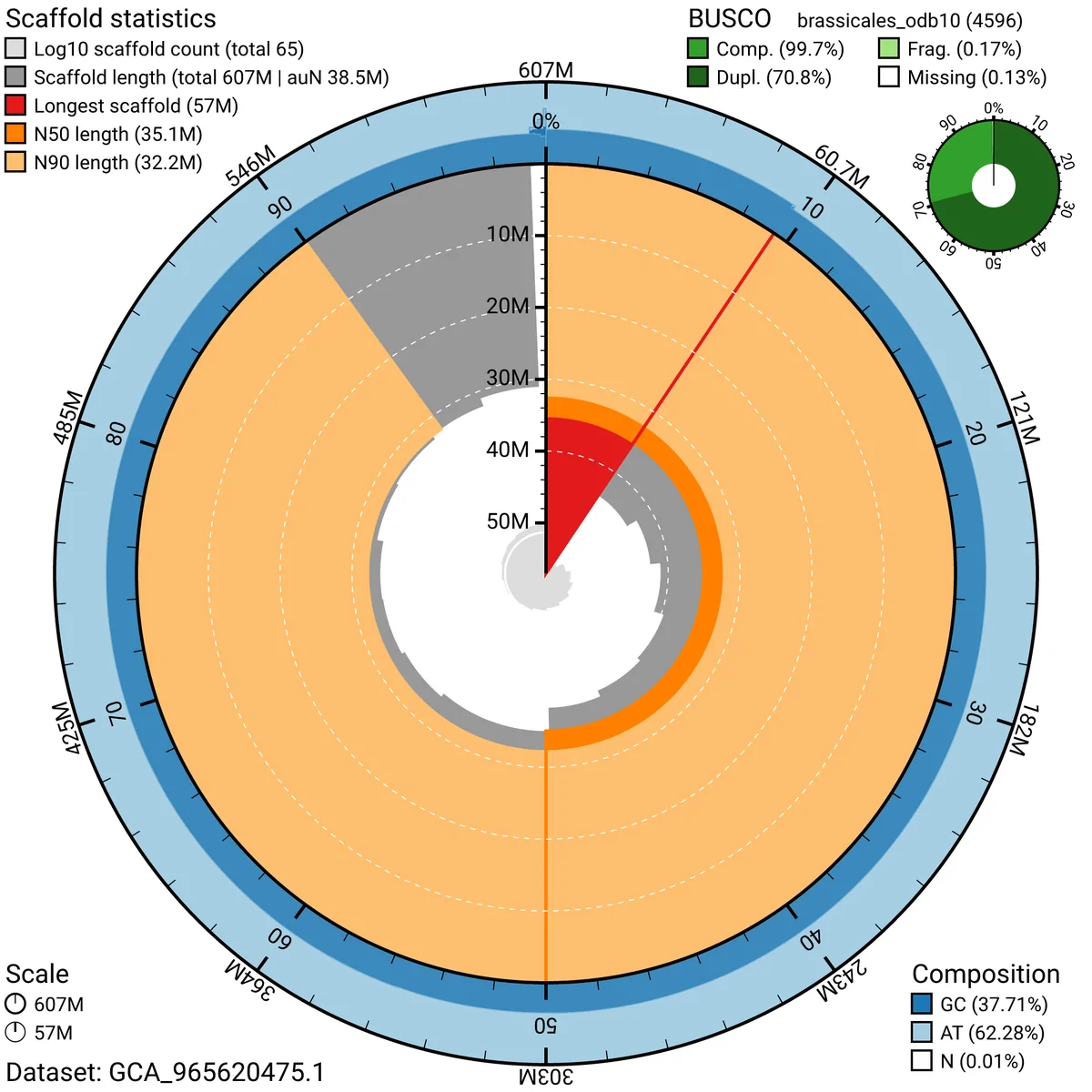
Assembly metrics for ddArmRust1.hap1.1, the haplotype 1 assembly. The BlobToolKit snail plot provides an overview of assembly metrics and BUSCO gene completeness. The circumference represents the length of the haplotype 1 assembly, and the main plot is divided into 1,000 bins around the circumference. The outermost blue tracks display the distribution of GC, AT, and N percentages across the bins. Scaffolds are arranged clockwise from longest to shortest and are depicted in dark grey. Scaffold length is shown on a linear radial scale, scaled to the longest scaffold. Red shading indicates the longest scaffold, and the deeper orange and pale orange shading represent the N50 and N90 lengths. The summary statistics include scaffold auN, the area under the scaffold Nx curve. The light grey spiral at the centre shows the cumulative scaffold count on a logarithmic scale. A summary of complete, fragmented, duplicated, and missing BUSCO genes in the brassicales_odb10 set is presented at the top right. The assembly can be explored interactively in the BlobToolKit viewer.

**Figure 6.**
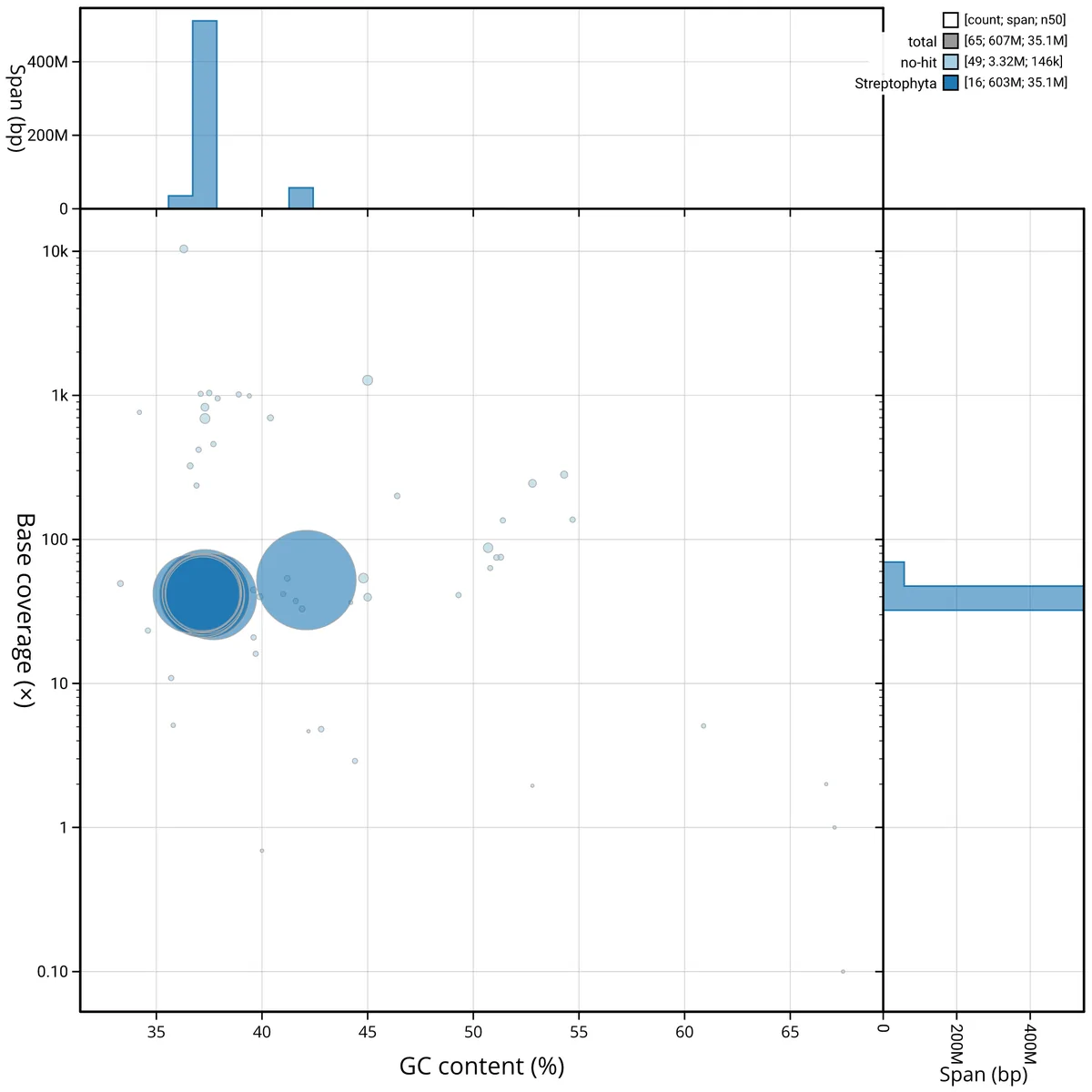
BlobToolKit blob plot for ddArmRust1.hap1.1, the haplotype 1 assembly. The plot shows base coverage (vertical axis) and GC content (horizontal axis). Circles represent scaffolds; marker area is proportional to scaffold length, and colour indicates taxonomic assignment. The histograms along the axes display the total length of sequences distributed across different levels of coverage and GC content. The assembly can be explored interactively in the BlobToolKit viewer.

Table 5 lists the assembly metric benchmarks adapted from Rhie *et al.*. (2021) and the Earth BioGenome Project (EBP) Report on Assembly Standards. The three-part EBP metric calculated for the haplotype 1 assembly is **6.C.Q66**.

**Table 5.** Earth BioGenome Project summary metrics for the *A. rusticana* assembly

**Measure**	**Value**	***Benchmark***
EBP summary (haplotype 1)	6.C.Q66	*6.C.Q40*
Contig N50 length	3.30 Mb	*> 1 Mb*
Scaffold N50 length	35.05 Mb	*= chromosome N50*
Consensus quality (QV)	Haplotype 1: 66.3; haplotype 2: 65.2; combined: 65.7	*≥ 40*
*k*-mer completeness	Haplotype 1: 81.70%; Haplotype 2: 81.61%; combined: 97.61%	*> 90%*
BUSCO	Haplotype 1: C:99.7% [S:28.9%, D:70.8%], F:0.2%, M:0.1%, n:4,596; Haplotype 2: C:99.7% [S:29.1%, D:70.6%], F:0.2%, M:0.2%, n:4,596	*S > 90%; D < 5%*
Percentage of assembly assigned to chromosomes	99.56%	*≥ 90%*

**Notes:** The EBP summary uses log10(Contig N50); chromosome-level (C) or log10(Scaffold N50); Q (Merqury QV). BUSCO v5.8.3 using the brassicales_odb10 lineage dataset: C=complete; S=single-copy; D=duplicated; F=fragmented; M=missing; n=orthologues.

### Genome annotation report

The *A. rusticana* genome assembly (GCA_965620475.1) was annotated by Ensembl at the European Bioinformatics Institute (EBI). The annotation comprises 74,743 transcripts from 40,675 protein-coding genes and 21,371 non-coding genes. The average transcript length is 2,072.69 bp, with an average of 1.20 coding transcripts per gene and 4.56 exons per transcript. For further information about the annotation, please refer to the annotation page on Ensembl.

### Data availability

European Nucleotide Archive: Armoracia rusticana, ddArmRust. Accession number PRJEB80112; https://identifiers.org/ena.embl/PRJEB80112. The genome sequence is released openly for reuse. The *A. rusticana* genome sequencing initiative is part of the Darwin Tree of Life Project ( PRJEB40665), the Sanger Institute Tree of Life Programme ( PRJEB43745) and AEGIS ( PRJEB80366). All raw sequence data and the haplotype assemblies have been deposited in INSDC databases. Raw data and assembly accession identifiers are reported in Table 2 and Table 3.

Pipelines used for genome assembly at the WSI Tree of Life are available at https://pipelines.tol.sanger.ac.uk/pipelines. Table 6 lists software versions used in this study.

**Table 6.** Software versions and sources used for *A. rusticana*

**Software**	**Version**	**Source**
BLAST	2.14.0	https://ftp.ncbi.nlm.nih.gov/blast/executables/blast+/
BlobToolKit	4.4.6	https://github.com/genomehubs/blobtoolkit
BlobTk (hosted BlobDir)	0.5.1	https://github.com/genomehubs/blobtk
BlobTk (snail and blob plot renderer)	0.8.3	https://github.com/genomehubs/blobtk
BUSCO	5.8.3; 6.1.0	https://gitlab.com/ezlab/busco
bwa-mem2	2.2.1	https://github.com/bwa-mem2/bwa-mem2
DIAMOND	2.1.8	https://github.com/bbuchfink/diamond
fasta_windows	0.2.4	https://github.com/tolkit/fasta_windows
FastK	1.2	https://github.com/thegenemyers/FASTK
GenomeScope	2.1.0	https://github.com/tbenavi1/genomescope2.0
Gfastats	1.3.6	https://github.com/vgl-hub/gfastats
Hifiasm	0.19.8-r603	https://github.com/chhylp123/hifiasm
HiGlass	1.13.4	https://github.com/higlass/higlass
MerquryFK	1.1.0-c1	https://github.com/thegenemyers/MERQURY.FK
Minimap2	2.28-r1209	https://github.com/lh3/minimap2
Oatk	0.9	https://github.com/c-zhou/oatk
MultiQC	1.14; 1.17 and 1.18	https://github.com/MultiQC/MultiQC
Nextflow	24.10.4	https://github.com/nextflow-io/nextflow
PretextSnapshot	0.0.6	https://github.com/sanger-tol/PretextSnapshot
PretextView	1.0.3	https://github.com/sanger-tol/PretextView
samtools	1.21	https://github.com/samtools/samtools
sanger-tol/ascc	0.1.0	https://github.com/sanger-tol/ascc
sanger-tol/blobtoolkit	v0.8.0	https://github.com/sanger-tol/blobtoolkit
sanger-tol/curationpretext	1.4.2	https://github.com/sanger-tol/curationpretext
Seqtk	1.4-r122	https://github.com/lh3/seqtk
Singularity	3.9.0	https://github.com/sylabs/singularity
TreeVal	1.1.1	https://github.com/sanger-tol/treeval
YaHS	1.2a.2	https://github.com/c-zhou/yahs

### Author information

- Members of the Royal Botanic Gardens Kew Genome Acquisition Lab
- Members of the Plant Genome Sizing Collective
- Members of the Darwin Tree of Life Barcoding collective
- Members of the Wellcome Sanger Institute Tree of Life Management, Samples and Laboratory team
- Members of Wellcome Sanger Institute Scientific Operations – Sequencing Operations
- Members of the Wellcome Sanger Institute Tree of Life Core Informatics team
- Members of the Tree of Life Core Informatics collective
- Members of the Darwin Tree of Life Consortium

### Wellcome Sanger Institute – Legal and Governance

The materials that have contributed to this genome note have been supplied by a Darwin Tree of Life Partner. The submission of materials by a Darwin Tree of Life Partner is subject to the **‘Darwin Tree of Life Project Sampling Code of Practice’**, which can be found in full on the Darwin Tree of Life website. By agreeing with and signing up to the Sampling Code of Practice, the Darwin Tree of Life Partner agrees they will meet the legal and ethical requirements and standards set out within this document in respect of all samples acquired for, and supplied to, the Darwin Tree of Life Project. Further, the Wellcome Sanger Institute employs a process whereby due diligence is carried out proportionate to the nature of the materials themselves, and the circumstances under which they have been/are to be collected and provided for use. The purpose of this is to address and mitigate any potential legal and/or ethical implications of receipt and use of the materials as part of the research project, and to ensure that in doing so we align with best practice wherever possible. The overarching areas of consideration are:

- Ethical review of provenance and sourcing of the material
- Legality of collection, transfer and use (national and international)

Each transfer of samples is further undertaken according to a Research Collaboration Agreement or Material Transfer Agreement entered into by the Darwin Tree of Life Partner, Genome Research Limited (operating as the Wellcome Sanger Institute), and in some circumstances, other Darwin Tree of Life collaborators.
