# Supplementary material for: The genome sequence of horse-radish, Armoracia rusticana G.Gaertn., B.Mey. & Scherb. (Brassicales: Brassicaceae)
Source: Wellcome Open Res. 2026 Sep 18;11:698. [Version 1] doi: 10.12688/wellcomeopenres.27846.1 (PMC13722856; doi:10.12688/wellcomeopenres.27846.1)
Supplement: Peer review history

# Reviewer report: Reviewer response for version 1

**DOI:** 10.21956/wellcomeopenres.30643.r172153

**Published:** 2026-10-30

**Authors:** Bartosz Ulaszewski

**Affiliations:** 1 Kazimierz Wielki University, Chodkiewicza, Bydgoszcz, Poland

This Genome Note provides information on the sequencing, assembly, and annotation of *Armoracia rusticann *a widespread species from the northern hemisphere. In my comments on this work, I’d like to use the mandatory questions as a starting points to discuss a few specific elements of the manuscript:

**1)** as a someone from East Europe, I'm happy that Darwin Tree of Life Consortium, finally picked this important cultural and culinary plant species. On the other hand from the ecological standpoint this species success of inhabiting most of the Northern Hemisphere is quite impressing but it comes with a cost, from my point of view some information on invasive potential of the plant [1] is missing in the manuscript;

**2)** the description of the laboratory protocols is the strongest point of this Genome Note. Each set is well and clearly described with attention to detail. Availability in a public repository (protocols.io) allows for smooth replication of the workflow;

**3)** As a bioinformatician, I'm pleased that the team behind this paper provided a very detailed description of the sequence assembly, from data curation to chromosome building and genome phasing. On the other hand, the annotation part of the manuscript lacks detail I know that is was build by Ensembl but if want to replicate the results it's unclear in the manuscript how the annotation was done and what datasets/protocols were used.

**4)** here I would like to also praise the extra effort made be the Team behind this paper on the precise choosing of the specimen for this study.

**5)** the results regarding genome size differences between flow cytometry, GenomeScope analysis, and the size of the final (phased) assembly are, in my opinion, correct when we consider the allotetraploidy of this species. The same applies to the high level of duplication.

 In brief, the paper is very well described, and with small adaptations, it would be very useful for other researchers on how to assemble a genome of a non-model plant species, from picking the right individual to wet-lab procedures, sequencing strategy, and the final genome assembly.

 Reference: Heinonen, M., & Åhlström, S. (2020). Horseradish in use and preserved in collections in Finland. Landraces, 32

Are the datasets clearly presented in a usable and accessible format, and the assembly and annotation available in an appropriate subject-specific repository?

Yes

Are sufficient details of the sequencing and extraction, software used, and materials provided to allow replication by others?

Yes

Are the rationale for sequencing the genome and the species significance clearly described?

Yes

Are the protocols appropriate and is the work technically sound?

Yes

Reviewer Expertise:

Plant genomics

I confirm that I have read this submission and believe that I have an appropriate level of expertise to confirm that it is of an acceptable scientific standard.

# Reviewer report: Reviewer response for version 1

**Published:** 2026-09-18

**Authors:** Automated Benchmarking

**Affiliations:** 1 Wellcome Sanger Institute, Hinxton, UK

As this article is a Genome Note, Automated Benchmarking has been used to determine whether the reported assembly passes four defined checks established by the Earth BioGenome Project: the raw and assembled data are publicly available, the Earth BioGenome Project three-part metric is met, the k-mer completeness exceeds 90%, and the BUSCO score passes the stated threshold. For Armoracia rusticana (ddArmRust1), all four checks passed. Data available: pass. ENA study PRJEB80112, raw-read accessions ERR13698296, ERR13698295, ERR13698294, ERR13670059, and ERR13670058, and assembly accessions GCA_965620475.1 and GCA_965620565.1 are public. Earth BioGenome Project three-part metric: pass. Haplotype 1 is reported as 6.C.Q66, meeting the 6.C.Q40 reference standard. k-mer completeness: pass. The combined haplotype assemblies have 97.61% k-mer completeness, exceeding the Earth BioGenome Project target of 90%. BUSCO: pass. Haplotype 1, which is proposed as the reference assembly for this species, has 99.7% complete BUSCOs (28.9% single-copy and 70.8% duplicated). BUSCO software version: 5.8.3; lineage dataset: brassicales_odb10. It meets the plant-specific target of greater than 90% complete BUSCOs. This decision uses values reported in the Genome Note and public accession records.

Reviewer Expertise:

NA

I confirm that I have read this submission and believe that I have an appropriate level of expertise to confirm that it is of an acceptable scientific standard.
